# Supplementary material for: Taxometer: Improving taxonomic classification of metagenomics contigs
Source: Nat Commun. 2024 Sep 27;15:8357. doi: 10.1038/s41467-024-52771-y (PMC11437175; doi:10.1038/s41467-024-52771-y)
Supplement: Supplementary file 1 — Supplementary Information [file 41467_2024_52771_MOESM1_ESM.pdf]

Supplementary materials for the paper "Taxometer: Improving taxonomic classification of metagenomics contigs"

List of Figures

1 Flat softmax hierarchical loss 3

2 Rhizosphere annotation and prediction quality for all domains 4

3 Benchmark of ZymoBIOMICS microbial community standard sample . 5

4 Benchmark of ZymoBIOMICS gut microbiome standard sample. . . . 6

5 Precision-recall curves for CAMI2 human microbiome 7

6 Precision-recall curves for CAMI2 marine and rhizosphere 8

7 Contribution of abundances and TNFs features to Taxometer performance 9

8 CAMI2 one-sample experiment 10

9 VAMB bins for taxonomic refinement 11

10 Predictions at different confidence levels of Kraken2, CAMI2 12

11 F-score and precision-recall curves at different confidence levels, CAMI2 Gastrointestinal 13

12 F-score and precision-recall curves at different confidence levels, CAMI2 Rhizosphere 14

13 Cross-validation evalution of Taxometer predictions 15

14 Precisions of Taxometer and classifiers for each dataset 16

15 Long-read datasets classification discrepancies 17

16 K-fold results for the long-read datasets 18

17 GPU runtimes for all datasets 19

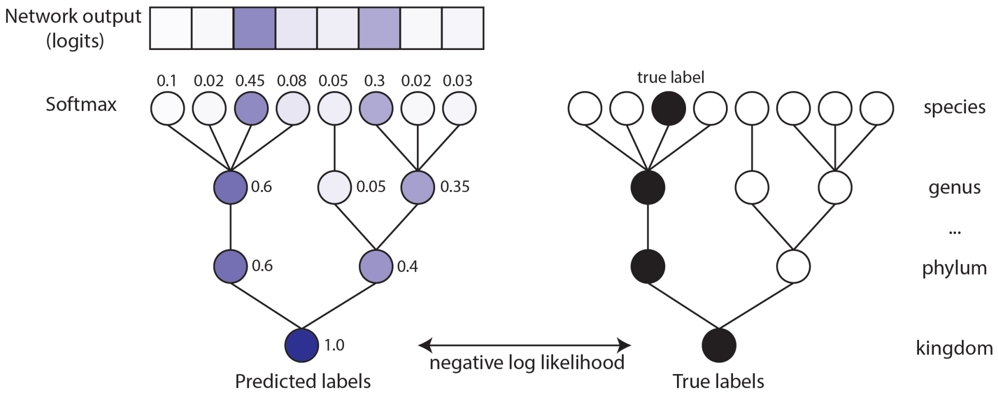

Supplementary Figure 1 Flat softmax hierarchical loss. The network output is a vector of N_l logits, where N_l is the number of leaves (species) on the taxonomic tree. The softmax likelihoods are recursively summed bottom up. The negative log likelihood is computed between the true and predicted labels on all taxonomic levels.

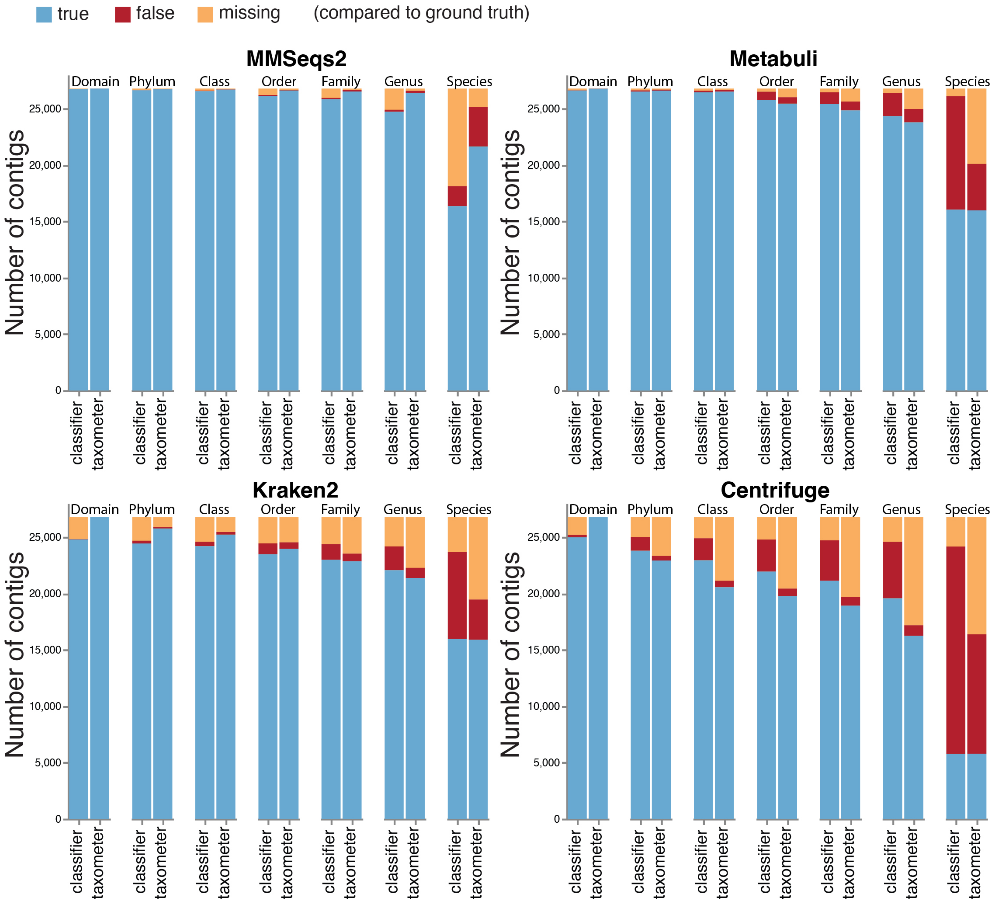

Supplementary Figure 2 Rhizosphere annotation quality and prediction quality for all domains. The number of true, false and missing annotations for four taxonomic classifiers and predictions of Taxometer trained on each classifier, compared to ground truth. Source data are provided as a Source Data file.

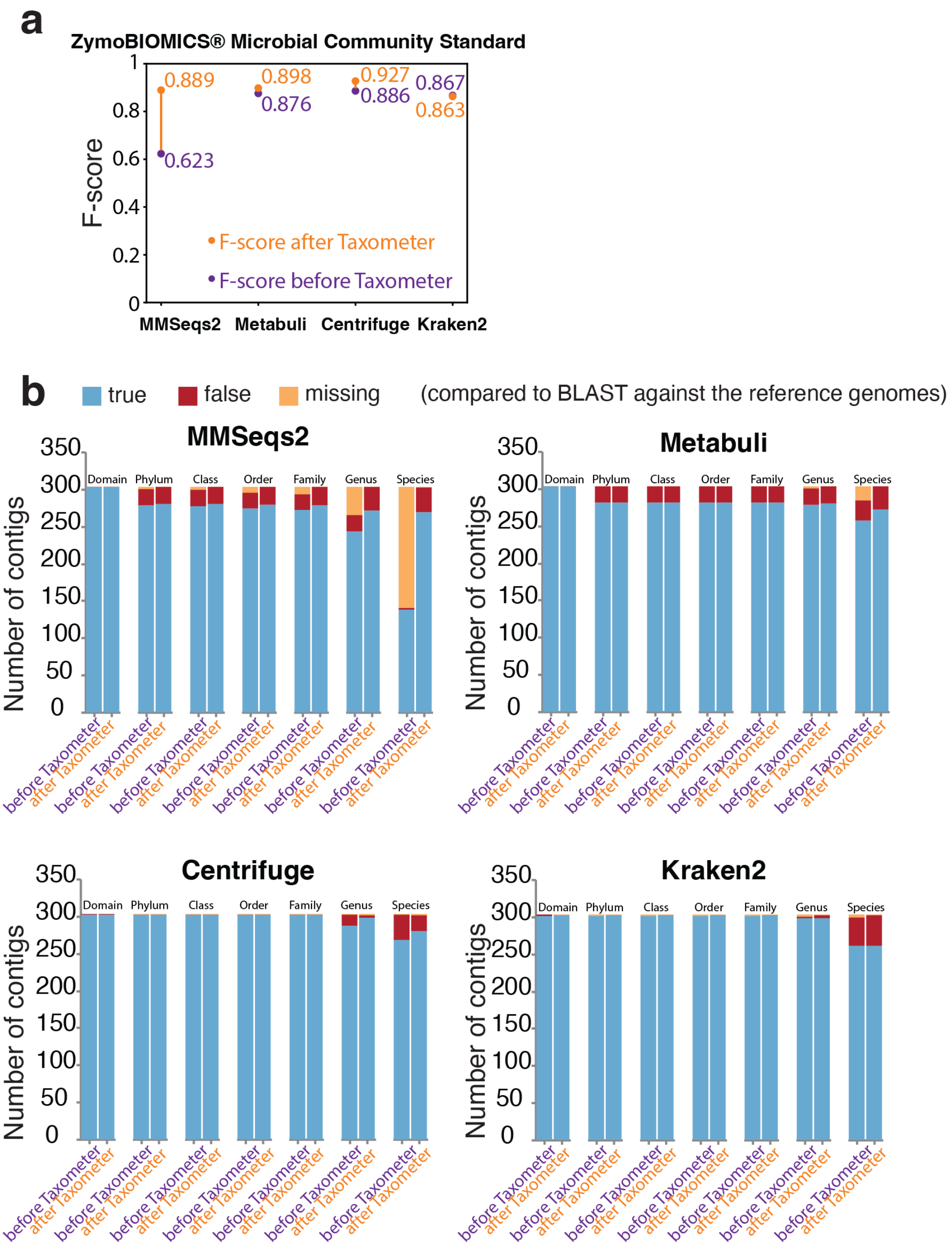

Supplementary Figure 3 Benchmark of ZymoBIOMICS microbial community standard sample a Taxonomic classifier annotations and Taxometer F-scores at species level, using results of BLAST to the reference genomes as gold standard. **b** The number of true, false and missing annotations of the same dataset for four taxonomic classifiers and predictions of Taxometer trained on each classifier, compared to the results of BLAST to the reference genomes. The score threshold value is 0.5. Source data are provided as a Source Data file.

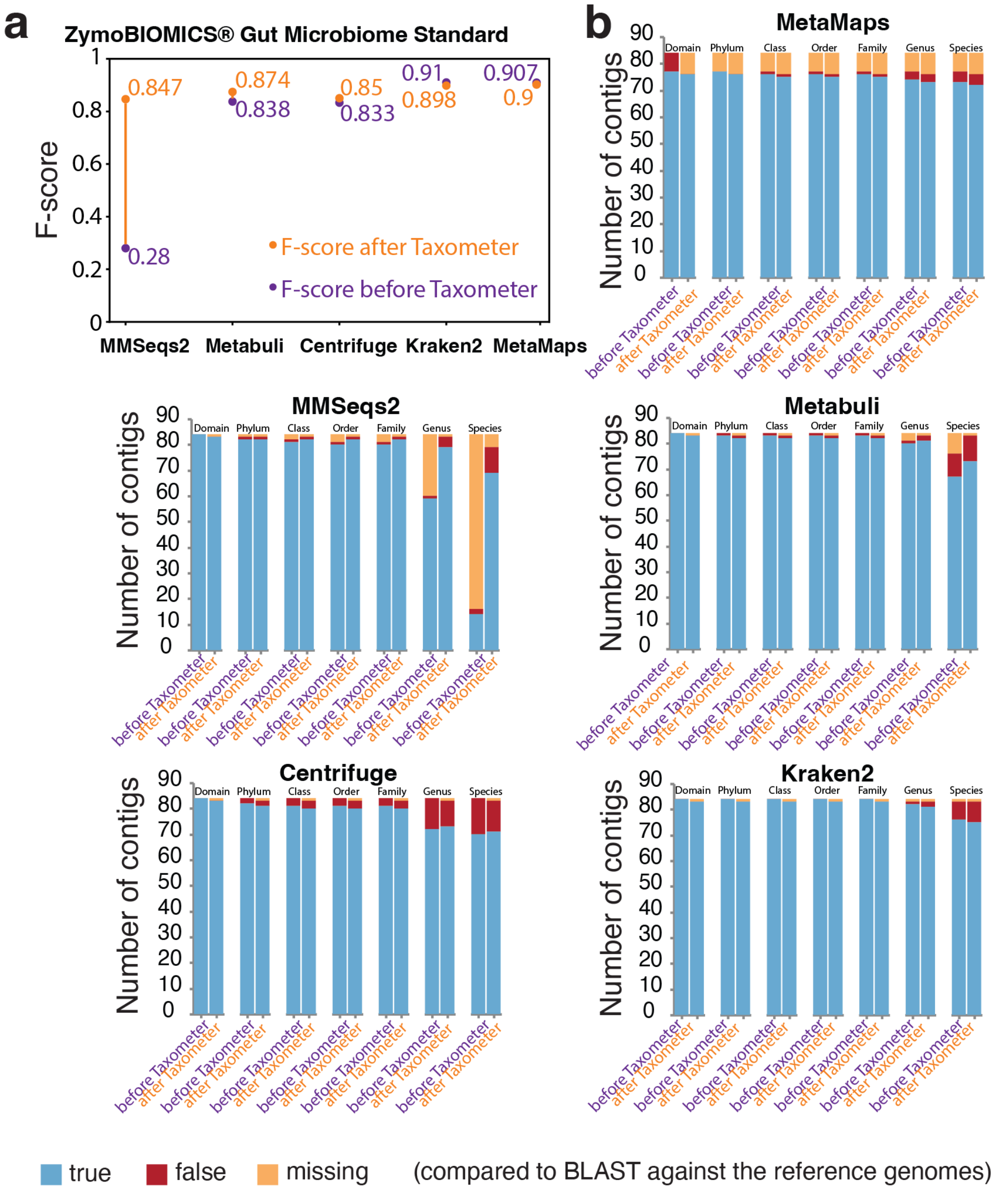

Supplementary Figure 4 Benchmark of ZymoBIOMICS gut microbiome standard sample. a Taxonomic classifier annotations and Taxometer F-scores at species level, using results of BLAST to the reference genomes as gold standard. **b** The number of true, false and missing annotations of the same dataset for four taxonomic classifiers and predictions of Taxometer trained on each classifier, compared to the results of BLAST to the reference genomes. The score threshold value is 0.5. Source data are provided as a Source Data file.

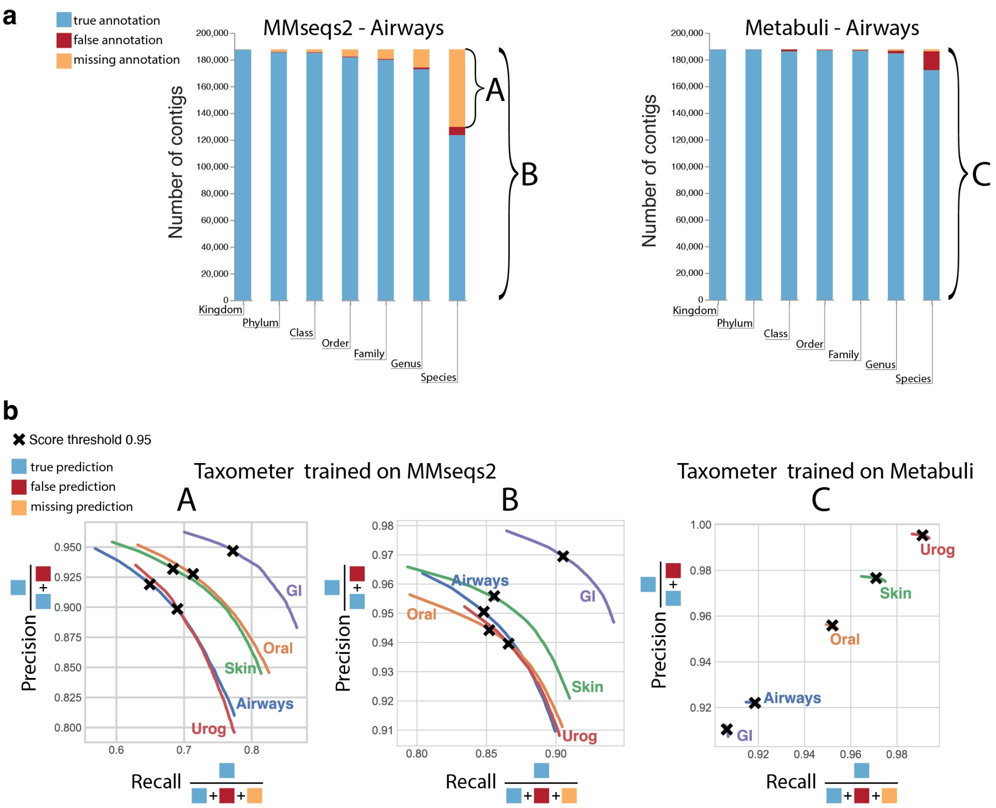

Supplementary Figure 5 Precision-recall curves for CAMI2 human microbiome. a, The number of true, false and missing MMseqs2 and Metabuli annotations for CAMI2 Airways dataset. **b,** Precision-recall curves for Taxometer scores in the range $[0.5, 1]$ for species labels calculated for A-labelled contigs with missing species-level MMseqs2 annotations; B- and C-labelled was based on all contigs. For A and B, Taxometer was trained on MMseqs2 annotations, and for C using Metabuli annotations. The cross marks the score threshold 0.95. Source data are provided as a Source Data file.

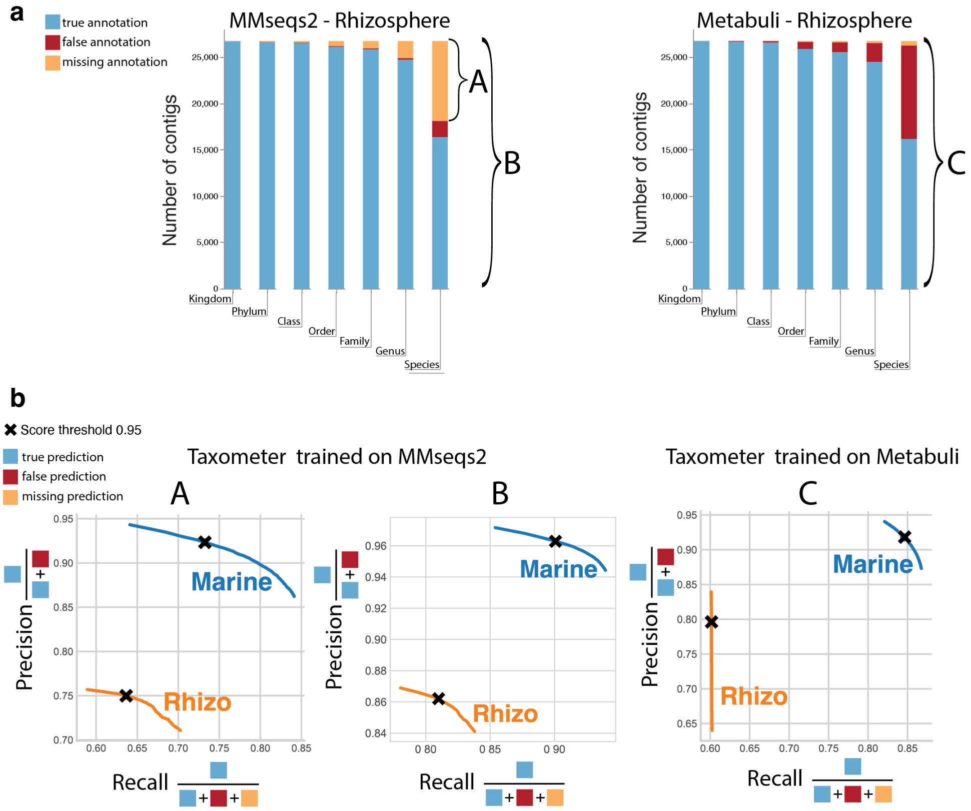

Supplementary Figure 6 Precision-recall curves for CAMI2 marine and rhizosphere. a, The number of true, false and missing MMseqs2 and Metabuli annotations for CAMI2 Rhizosphere dataset. **b,** Precision-recall curves for Taxometer scores in the range [0.5, 1] for species labels calculated for A-labelled contigs with missing species-level MMseqs2 annotations; B- and C- labelled was based on all contigs. For A and B, Taxometer is trained on MMseqs2 annotations, for C, on Metabuli annotations. The cross marks the score threshold 0.95. Source data are provided as a Source Data file.

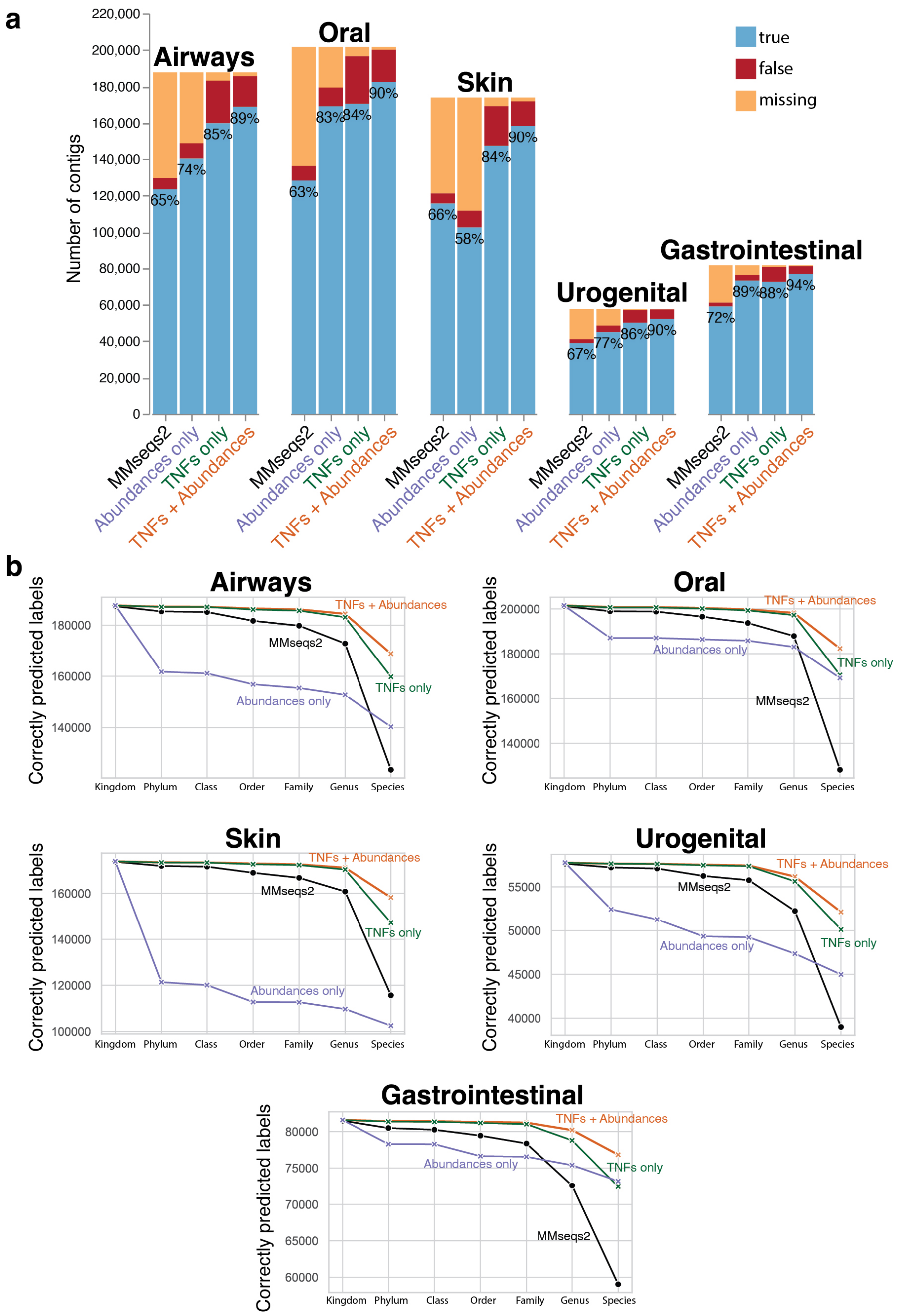

Supplementary Figure 7 Contribution of abundances and TNFs features to Taxometer performance. a, Species-level predictions compared to ground truth with score threshold 0.5, CAMI2 human microbiome short-read dataset. **b,** Number of correctly predicted labels on each taxonomic level. Source data are provided as a Source Data file.

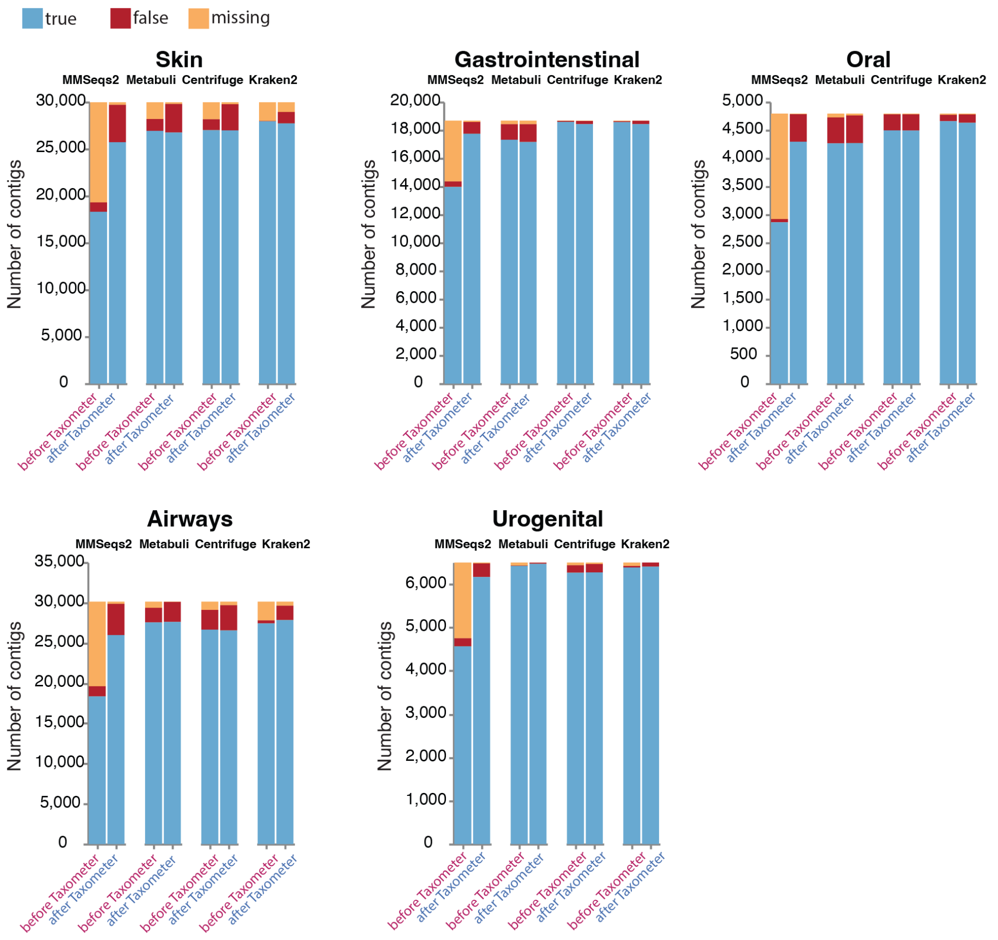

Supplementary Figure 8 CAMI2 one-sample experiment. Performance on CAMI2 human microbiome datasets when only using contigs from one sample in each dataset. The score threshold is 0.5. Source data are provided as a Source Data file.

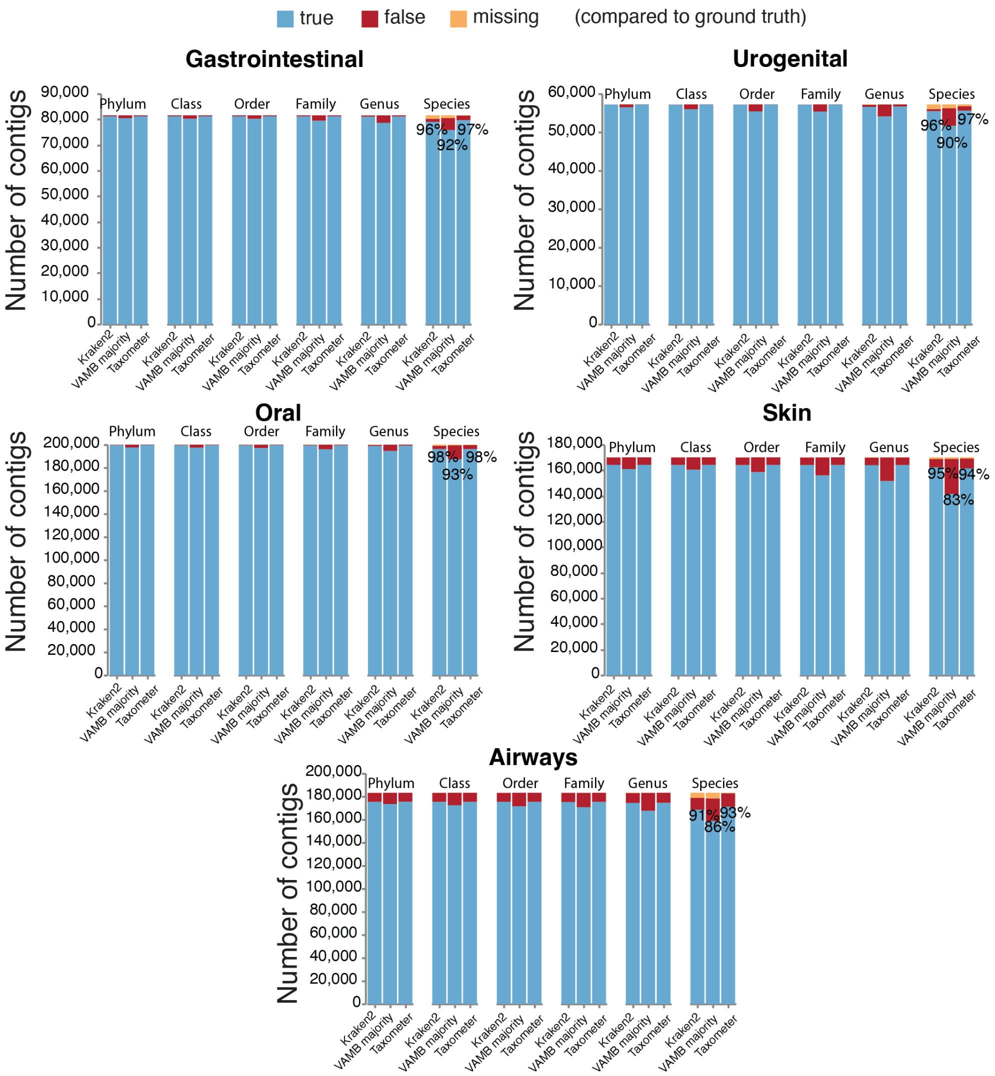

Supplementary Figure 9 VAMB bins for taxonomic refinement. The number of true, false and missing annotations for CAMI2 human microbiome datasets and Kraken2. Compared are: Kraken2 annotations, VAMB bin majority annotations, Taxometer refinement of Kraken2. Source data are provided as a Source Data file.

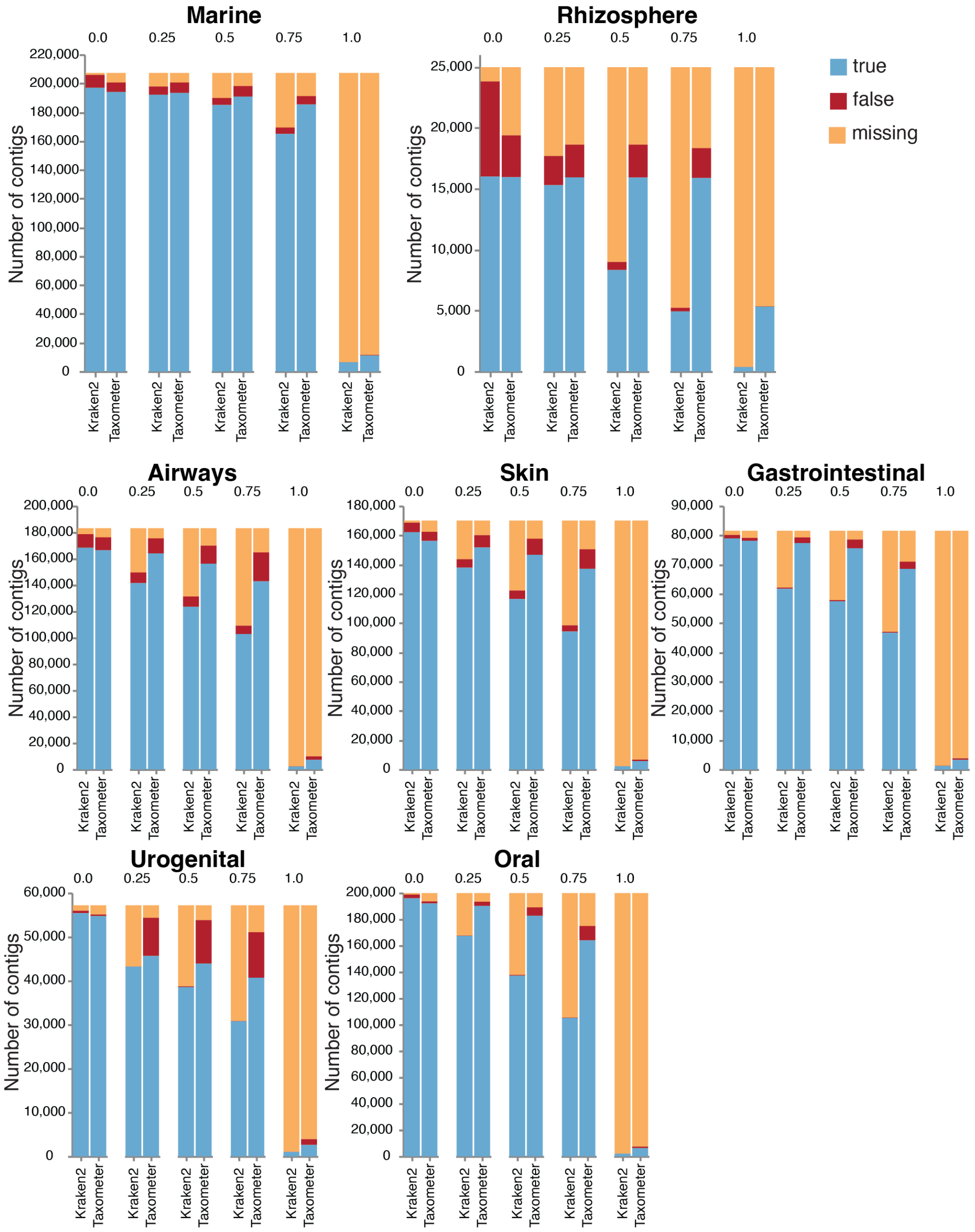

Supplementary Figure 10 Predictions at different confidence levels of Kraken2, CAMI2 True, false and missing labels returned by Kraken2 configured with different confidence levels and the corresponding Taxometer predictions. Source data are provided as a Source Data file.

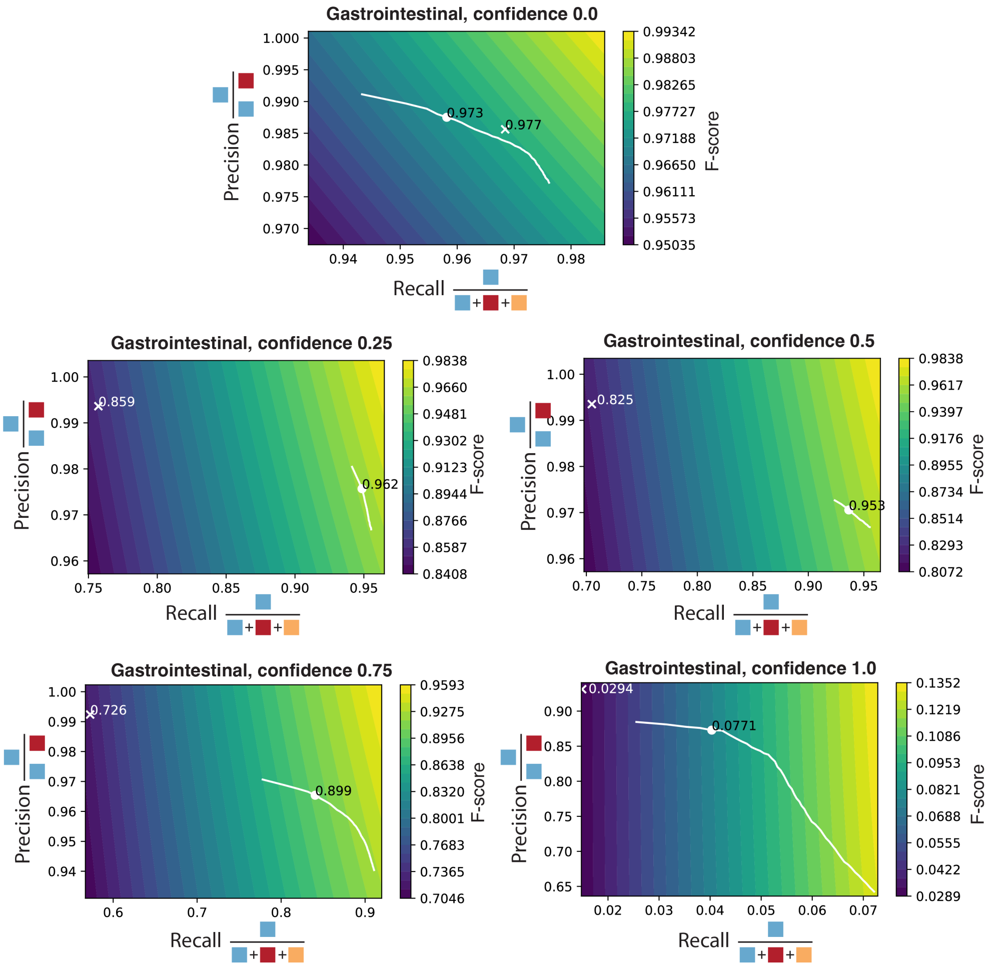

Supplementary Figure 11 F-score and precision-recall curves at different confidence levels, CAMI2 Gastrointestinal The color scale shows the F1-score for the given recall (x-axis) and precision (y-axis). The white circle marks the point with the Taxometer threshold 0.95. The cross marks the point of the precision and the recall of the Kraken2 classifier results. The white line shows the Taxometer precision-recall curve. Source data are provided as a Source Data file.

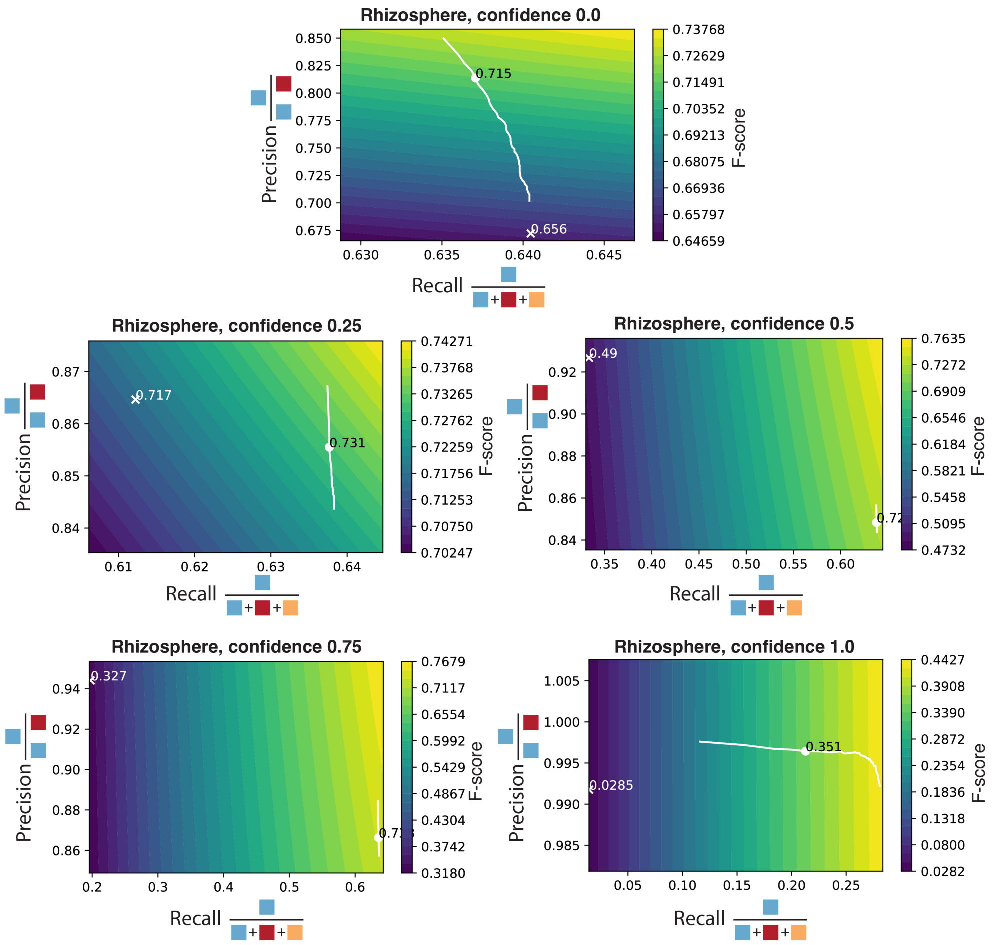

Supplementary Figure 12 F-score and precision-recall curves at different confidence levels, CAMI2 Rhizosphere The color scale shows the F1-score for the given recall (x-axis) and precision (y-axis). The white circle marks the point with the Taxometer threshold 0.95. The cross marks the point of the precision and the recall of the Kraken2 classifier results. The white line shows the Taxometer precision-recall curve. Source data are provided as a Source Data file.

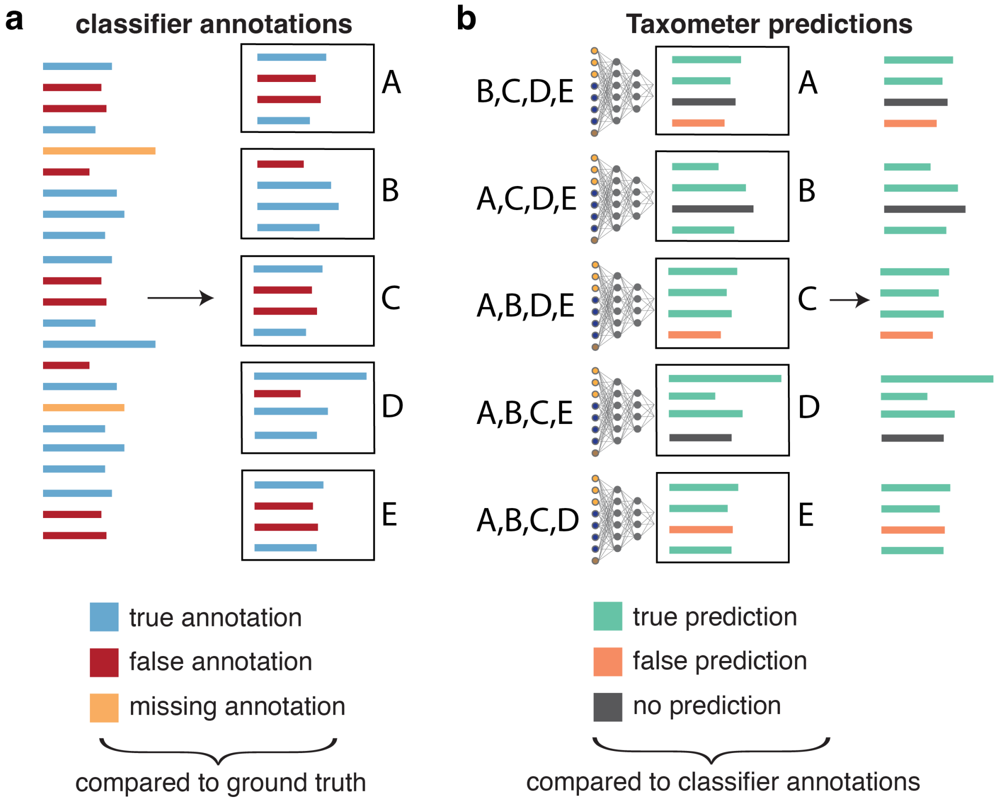

Supplementary Figure 13 Cross-validation evalution of Taxometer predictions. a, Classifier annotations are splitted into 5 folds. **b,** The annotations for contigs from each fold are predicted using Taxometer, trained on the remaining four. The results are then compared to the classifier annotations.

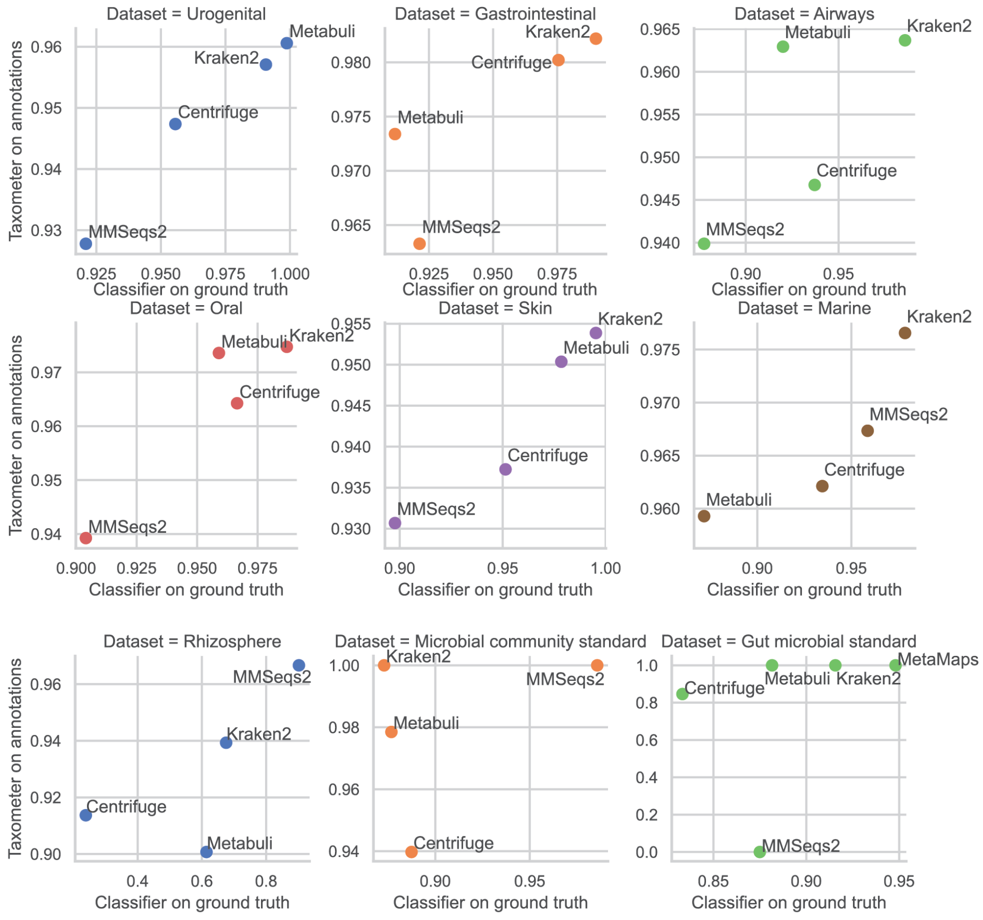

Supplementary Figure 14 Precisions of Taxometer and classifiers for each dataset. X-axis is precision of a taxonomic classifier compared to ground truth, Y-axis is precision of Taxometer predicting the taxonomic classifier annotations, score threshold 0.5, on a subplot per dataset. Source data are provided as a Source Data file.

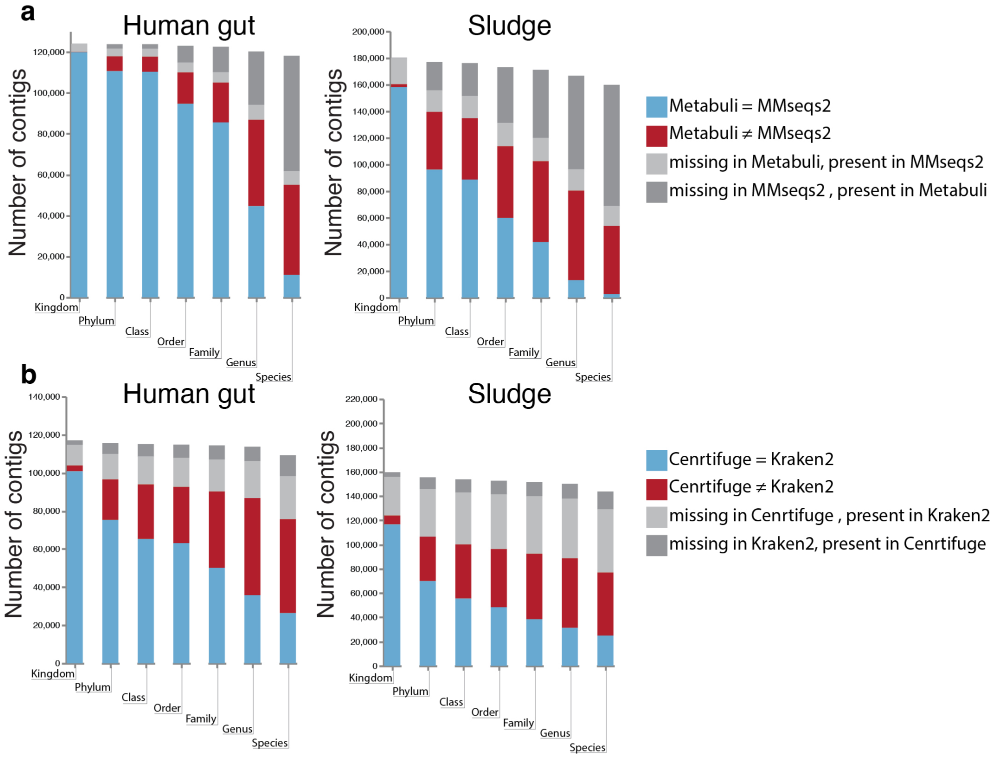

Supplementary Figure 15 Long-read datasets classification discrepancies. a, Discrepancies between long-read contigs annotations of GTDB classifiers, MMseqs2 and Metabuli, on all taxonomic levels. **b,** Discrepancies between long-read contigs annotations of NCBI classifiers, Kraken2 and Centrifuge, on all taxonomic levels. Source data are provided as a Source Data file.

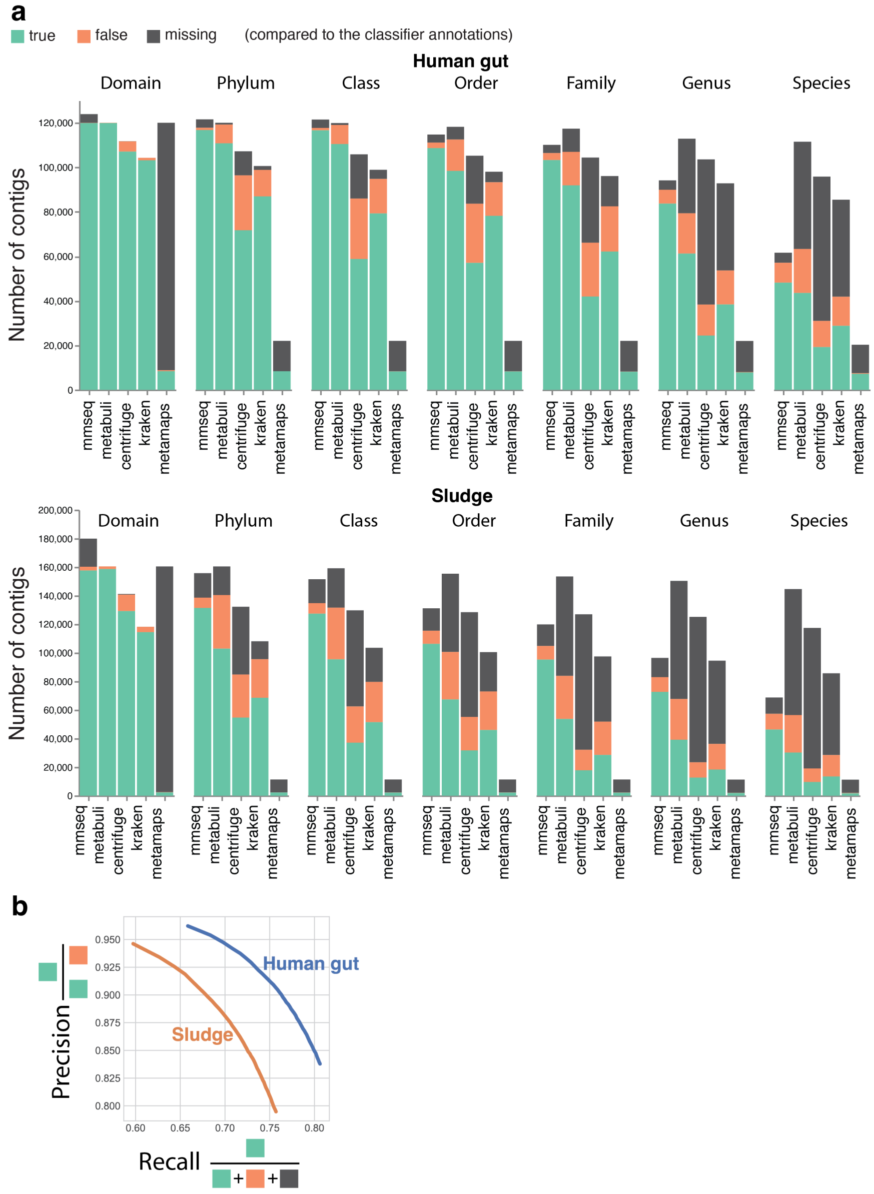

Supplementary Figure 16 K-fold results for the long-read datasets. a, The number of true, false and no predictions of Taxometer for four taxonomic classifiers, compared to classifiers annotations, long-read datasets (Human Gut, Sludge), all taxonomic levels. **b,** Precision-recall curves for Taxometer scores in the range $[0.5, 1]$ for species labels, long-read datasets, MMseqs2 classifier. Source data are provided as a Source Data file.

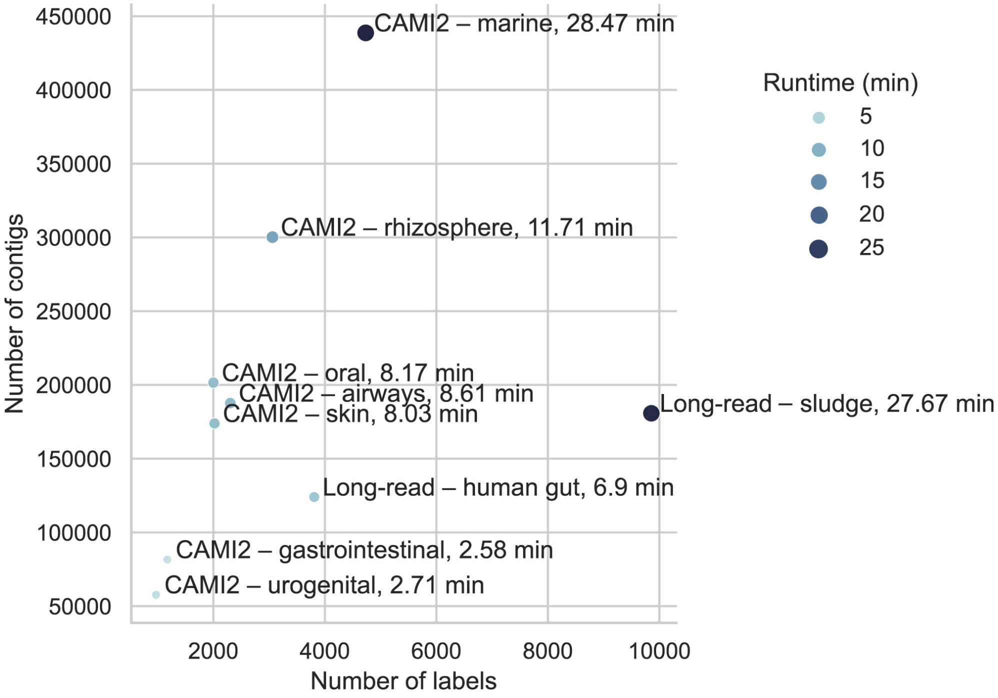

Supplementary Figure 17 GPU runtimes for all datasets. X-axis is the number of leaf (species) labels in the taxonomic tree constructed from the classifier annotations for a dataset. Y-axis is the number of contigs in a dataset. The size and hue of a point varies according to the runtime on 1 GPU, in minutes. Source data are provided as a Source Data file.
