# Supplementary material for: Taxometer: Improving taxonomic classification of metagenomics contigs
Source: Nat Commun. 2024 Sep 27;15:8357. doi: 10.1038/s41467-024-52771-y (PMC11437175; doi:10.1038/s41467-024-52771-y)
Supplement: Supplementary file 3 — Reporting Summary [file 41467_2024_52771_MOESM3_ESM.pdf]

nature portfolio

Corresponding author(s): Simon Rasmussen

Last updated by author(s): 29-08-2024

Reporting Summary

Nature Portfolio wishes to improve the reproducibility of the work that we publish. This form provides structure for consistency and transparency in reporting. For further information on Nature Portfolio policies, see our Editorial Policies and the Editorial Policy Checklist.

Statistics

For all statistical analyses, confirm that the following items are present in the figure legend, table legend, main text, or Methods section.

n/a	Confirmed	
☐	☒	The exact sample size (*n*) for each experimental group/condition, given as a discrete number and unit of measurement
☒	☐	A statement on whether measurements were taken from distinct samples or whether the same sample was measured repeatedly
☒	☐	The statistical test(s) used AND whether they are one- or two-sided<br>*Only common tests should be described solely by name; describe more complex techniques in the Methods section.*
☒	☐	A description of all covariates tested
☒	☐	A description of any assumptions or corrections, such as tests of normality and adjustment for multiple comparisons
☒	☐	A full description of the statistical parameters including central tendency (e.g. means) or other basic estimates (e.g. regression coefficient) AND variation (e.g. standard deviation) or associated estimates of uncertainty (e.g. confidence intervals)
☒	☐	For null hypothesis testing, the test statistic (e.g. *F*, *t*, *r*) with confidence intervals, effect sizes, degrees of freedom and *P* value noted<br>*Give P values as exact values whenever suitable.*
☒	☐	For Bayesian analysis, information on the choice of priors and Markov chain Monte Carlo settings
☒	☐	For hierarchical and complex designs, identification of the appropriate level for tests and full reporting of outcomes
☐	☒	Estimates of effect sizes (e.g. Cohen's *d*, Pearson's *r*), indicating how they were calculated

Our web collection on statistics for biologists contains articles on many of the points above.

Software and code

Policy information about availability of computer code

Data collection	No software used as the publicly available data were use
Data analysis	Custom software presented in the submitted article: https://github.com/RasmussenLab/vamb/blob/taxometer release/ README Taxometer.md<br><br>Other software packages:<br>Centrifuge v1.0.4<br>Kraken2 v2.1.3<br>Metabuli v1.0.1<br>MMseqs v.7e2840<br>metaMDBG v. b55df39<br>minimap2 v.2.24<br>pycoverm v.0.6.0<br>bwa-mem v.0.7.15<br>samtools v.1.14<br>MetaMaps v.33d2e<br>gtdb_to_taxdump v.1.0.9<br>NCBI-BLAST v.2.15.0<br>pytorch v.1.13.1<br>CUDA v.11.7.99

GTDK-tk v.2.4.0

For manuscripts utilizing custom algorithms or software that are central to the research but not yet described in published literature, software must be made available to editors and reviewers. We strongly encourage code deposition in a community repository (e.g. GitHub). See the Nature Portfolio guidelines for submitting code & software for further information.

Data

Policy information about availability of data

All manuscripts must include a data availability statement. This statement should provide the following information, where applicable:

- Accession codes, unique identifiers, or web links for publicly available datasets
- A description of any restrictions on data availability
- For clinical datasets or third party data, please ensure that the statement adheres to our policy

We used synthetic CAMI2 datasets from five human microbiomes (Airways, Oral, Skin, Urogenital, and Gastrointestinal) and two environmental microbiomes (Marine and Rhizosphere): https://frl.publisso.de/data/frl:642S521/plant associated/ https://frl.publisso.de/data/frl:642S521/marine/ https://frl.publisso.de/data/frl:642S518/

We used two real PacBio HiFi long-read datasets, a 'human gut' dataset from human stool sample https://downloads.pacbcloud.com/public/dataset/Sequel-IIe-202104/metagenomics/ and a 'sludge' dataset from anaerobic digestion reactor sludge (ENA accessions ERR10905741-ERR10905743).

We used the two ZymoBIOMICS Microbial Community Standards: ZymoBIOMICS Microbial Community Standard and ZymoBIOMICS Gut Microbiome Standard from https://zymoresearch.eu/collections/zymobiomics-microbial-community-standards/products/zymobiomics-gut-microbiome-standard and https://zymoresearch.eu/collections/zymobiomics-microbial-community-standards/products/zymobiomics-microbial-community-standard. All data generated in this study are available as Source Data.

Research involving human participants, their data, or biological material

Policy information about studies with human participants or human data. See also policy information about sex, gender (identity/presentation), and sexual orientation and race, ethnicity and racism.

Reporting on sex and gender	The BioCollective pooled gut microbiome sample is used, the participants information is not available
Reporting on race, ethnicity, or other socially relevant groupings	The BioCollective pooled gut microbiome sample is used, the participants information is not available
Population characteristics	The BioCollective pooled gut microbiome sample is used, the participants information is not available
Recruitment	The BioCollective pooled gut microbiome sample is used, the participants information is not available
Ethics oversight	The BioCollective pooled gut microbiome sample is used, the participants information is not available

Note that full information on the approval of the study protocol must also be provided in the manuscript.

Field-specific reporting

Please select the one below that is the best fit for your research. If you are not sure, read the appropriate sections before making your selection.

☒ Life sciences ☐ Behavioural & social sciences ☐ Ecological, evolutionary & environmental sciences

For a reference copy of the document with all sections, see nature.com/documents/nr-reporting-summary-flat.pdf

Life sciences study design

All studies must disclose on these points even when the disclosure is negative.

Sample size	We used the publicly available datasets with the size range of 50 000-450 000. Datasets are either common benchmarks (CAMI2), or the long-read datasets of sufficient complexity chosen by representing different environments, or the synthetic microbiome data that were publicly available.
Data exclusions	No data exclusions were made
Replication	The main results are reproduced across the 11 different datasets presented in the study

Randomization	The hyperparameter tuning was performed using the few synthetic datasets and applied to the real datasets (long read human gut and sludge)
Blinding	Blinding was not possible due to the use of public datasets.

Reporting for specific materials, systems and methods

We require information from authors about some types of materials, experimental systems and methods used in many studies. Here, indicate whether each material, system or method listed is relevant to your study. If you are not sure if a list item applies to your research, read the appropriate section before selecting a response.

Materials & experimental systems

n/a	Involved in the study
☒	☐ Antibodies
☒	☐ Eukaryotic cell lines
☒	☐ Palaeontology and archaeology
☒	☐ Animals and other organisms
☒	☐ Clinical data
☒	☐ Dual use research of concern
☒	☐ Plants

Methods

n/a	Involved in the study
☒	☐ ChIP-seq
☒	☐ Flow cytometry
☒	☐ MRI-based neuroimaging

Plants

Seed stocks	*Report on the source of all seed stocks or other plant material used. If applicable, state the seed stock centre and catalogue number. If plant specimens were collected from the field, describe the collection location, date and sampling procedures.*
Novel plant genotypes	*Describe the methods by which all novel plant genotypes were produced. This includes those generated by transgenic approaches, gene editing, chemical/radiation-based mutagenesis and hybridization. For transgenic lines, describe the transformation method, the number of independent lines analyzed and the generation upon which experiments were performed. For gene-edited lines, describe the editor used, the endogenous sequence targeted for editing, the targeting guide RNA sequence (if applicable) and how the editor was applied.*
Authentication	*Describe any authentication procedures for each seed stock used or novel genotype generated. Describe any experiments used to assess the effect of a mutation and, where applicable, how potential secondary effects (e.g. second site T-DNA insertions, mosiacism, off-target gene editing) were examined.*
